# Arbuscular Mycorrhizal Fungi Increase Nutritional Quality of Soilless Grown Lettuce while Overcoming Low Phosphorus Supply

**DOI:** 10.3390/foods11223612

**Published:** 2022-11-12

**Authors:** Fatjon Cela, Luciano Avio, Tommaso Giordani, Alberto Vangelisti, Andrea Cavallini, Alessandra Turrini, Cristiana Sbrana, Alberto Pardossi, Luca Incrocci

**Affiliations:** 1 Department of Agriculture, Food and Environment, University of Pisa, Via del Borghetto 80, 56124 Pisa, Italy; 2 Interdepartmental Research Center Nutrafood—Nutraceuticals and Food for Health, University of Pisa, 56124 Pisa, Italy; 3 CNR, Institute of Agricultural Biology and Biotechnology, Via Moruzzi 1, 56124 Pisa, Italy

**Keywords:** antioxidant capacity, biostimulant, *Funneliformis mosseae*, *Lactuca sativa* L. cv Salinas, leaf gas exchanges, mineral nutrition, nutraceuticals

## Abstract

Lettuce is widely used for its healthy properties, and it is of interest to increase them with minimal environmental impact. The purpose of this work was to evaluate the effect of the arbuscular mycorrhizal fungus (AMF) *Funneliformis mosseae* in lettuce plants (*Lactuca sativa* L. cv. Salinas) cultivated in a soilless system with sub-optimal phosphorus (P) compared with non-inoculated controls at two different P concentrations. Results show that lettuce inoculation with the selected AMF can improve the growth and the nutritional quality of lettuce even at sub-optimal P. Leaf content of chlorophylls, carotenoids, and phenols, known as important bioactive compounds for human health, was higher in mycorrhizal lettuce plants compared with non-mycorrhizal plants. The antioxidant capacity in AMF plants showed higher values compared with control plants grown at optimal P nutrition level. Moreover, leaf gas exchanges were higher in inoculated plants than in non-inoculated ones. Nitrogen, P, and magnesium leaf content was significantly higher in mycorrhizal plants compared with non-mycorrhizal plants grown with the same P level. These findings suggest that *F. mosseae* can stimulate plants growth, improving the nutritional quality of lettuce leaves even when grown with sub-optimal P concentration.

## 1. Introduction

Lettuce (*Lactuca sativa* L.) is one of the most important leaf vegetables, largely consumed because of its healthy properties, which are attributed to a large content of fiber and antioxidant compounds, such as vitamins C and E, carotenoids, and polyphenols [1,2]. Other pigments such as chlorophylls can contribute to both the sensory and health-promoting properties of lettuce [2,3]. There is a growing interest for soilless cultivation of lettuce under greenhouses, which allows higher productivity and better leaf quality as a result of a strict control of environmental and nutritional conditions [4,5].

In soilless culture, the phosphorus (P) concentration of the nutrient solution (NS) normally ranges from 1 to 2 mol m^−3^, which is almost 100 times higher than the P concentration in soil [6]. This essential element is limited and not renewable, and the actual rate of its extraction from phosphate rocks is higher than the rate of its replenishment [7,8]. In addition, large use of P may result in significant environmental issues, while P deficiency is leading to severe reduction in crop yield and vegetable quality, as observed in soilless culture systems [9,10].

Arbuscular mycorrhizal fungi (AMF) are important beneficial soil microorganisms establishing mutualistic associations with most food crops. These fungi enhance plant growth and mineral nutrition, especially increasing the uptake of P and receiving in exchange plant carbon compounds [11,12]. AMF obtain P by a fine network of extraradical hyphae, which, spreading in soil beyond root explored zones, supply up to 100% of plant P requirements in low P soil, through a fine modulation of plant root P uptake [13]. AMF facilitate host plants to grow vigorously under low P conditions and other stressful conditions including drought, salinity, herbivory, temperature, heavy metals, and diseases [14,15,16,17,18], triggering plant alterations at the physiological, metabolic, and molecular level [19] which lead to the accumulation of a considerable number of secondary metabolites in host plants.

Therefore, AMF inoculation, which is increasingly considered as a promising tool to trigger the synthesis of bioactive compounds in plants and for the production of healthy foods [20], could be usefully combined with soilless cultivation systems in order to reduce P utilization, possibly enhancing the quality and productivity of crops. Actually, studies investigating the effects of AMF inoculation on soilless grown lettuce [21,22,23] reported contrasting results. For instance, some authors [21,22,23] showed that AMF enhanced lettuce growth and nutritional quality, while others [24] found no differences in leaf production and macronutrient content in lettuce plants inoculated and not inoculated with AMF. These contrasting results are probably due to the different fungal isolates and lettuce cultivars utilized. In particular, the lettuce cv. Salinas represents an important commercial crop, which is highly mycorrhizal and positively responds to P level in soil [25]. 

The aim of this study was to assess the effects induced by an isolate of the AMF species *Funneliformis mosseae* on colonization level, plant growth, leaf gas exchange, content of mineral nutrients, total chlorophylls, carotenoids, total phenols, and antioxidant capacity in lettuce (cv. Salinas) plants grown in a soilless system with optimal (1.0 mM) or reduced (0.5 mM) P concentration in NS.

## 2. Materials and Methods

### 2.1. Biological Materials

The experiment was conducted in Pisa, Italy (latitude 43°43′ N, longitude 10°23′ E), using a crisphead lettuce (*Lactuca sativa* L. var. *capitata*) cv. Salinas.

The fungal isolate used was *Funneliformis mosseae* (T. H. Nicolson and Gerd.) C. Walker and A. Schüssler, isolate IMA1, maintained for several multiplication cycles under identical growth conditions at the laboratory of Microbiology, Department of Agricultural, Food and Agro-Environmental Sciences, University of Pisa, Italy. A mycorrhizal inoculum potential (MIP) bioassay [26] was performed to assess the activity of the AMF using *Cichorium intybus* L. as test plant: MIP was 30–40%. 

### 2.2. Growing System and Nutrient Solution

Plants were grown in growth chambers with the following climatic conditions: temperature 25 °C ± 2.5; relative humidity 65% ± 15; daily cumulated global radiation 10.80 MJ m^−2^ with a photosynthetic photon flux density (PPFD) of 250 µmoles m^−2^ s^−1^; photoperiod of 16 h day^−1^. Irradiance was measured using a portable radiometer (model LI-185B; LI-COR Inc., Lincoln, NE, USA) equipped with a quantum sensor (LI-190SB) to give PPFD in μmol photons m^−2^ s^−1^. Artificial light was provided by LED lamps (Futura, C-LED, Imola, Italy) having the following spectrum: 50% red (620–680 nm), 33% blue (420–480 nm), 17% green (520–560 nm). The light spectrum was measured with a spectrometer (FLAME-T-XR1-ES S/N: FLMT07829, Ostfildern, Germany). 

Plants were supplied with the NS prepared adding appropriate amounts of technical-grade inorganic salts to tap water. Phosphorus was added as KH_2_PO_4_, at optimal (1.0 mM [27,28,29,30] or reduced (0.5 mM) concentration. In order to maintain the same potassium concentration and electroneutrality in both NSs, the lower concentration of KH_2_PO_4_ (0.5 mM) in the low P NS was compensated by adding 0.25 mM K_2_SO_4_.

The concentration of other nutritive elements in both NSs were the following: 12.0 mM N-NO_3_; 0.5 mM N-NH_4_; 4.5 mM Ca; 2.0 mM Mg; 1.7 mM Na; 1.0 mM Cl; 30.0 µM Fe; 25.0 µM B; 10.0 µM Mn; 16.2 µM Zn; 3.0 µM Cu; 1.0 µM Mo. The pH and electrical conductivity (EC) of NSs were 5.5 and 2.4 dS m^−1^, respectively.

### 2.3. Experimental Design and Plant Inoculation

The experiment was arranged as a completely randomized design with three treatments, consisting of non-inoculated (or control) plants grown with optimal (HPC, High Phosphorus Control) or reduced (LPC, Low Phosphorus Control) P concentration in the NS, and plants inoculated with *F. mosseae* and grown with 0.5 mM P concentration (LPM, Low Phosphorus Mycorrhizal). Each treatment was applied to eight plants: one plant represented one replicate.

After sterilized lettuce seeds’ germination in Petri dishes, AMF inoculation was performed on selected seedlings in polystyrene plug trays. One lettuce seedling was transferred in each 16 mL cell, previously filled with 15 mL of crude inoculum, comprised of spores, mycelium, and fine colonized roots. Control plants were mock inoculated, using a steam-sterilized crude inoculum (at 121 °C for 30 min, on two consecutive days), and then each mock-inoculated plant was supplied with 4 mL of a sieved soil eluate (through a 50 μm pore diameter sieve and through Whatman No. 1 paper), obtained using living AMF inoculum, to ensure a common microbiota. All seedlings were grown in the growth chamber as described above and irrigated daily with approximately 2–5 mL of a low P nutrient solution.

After four weeks, each seedling was transplanted in a 1 L pot filled with calcined clay (OILDRI, Chicago, IL, USA) and inoculated again with AMF, adding the crude inoculum at the dose of 10% (*v*/*v*). Inside the growth chamber, the position of each plant was changed every two days in order to overcome the chamber effect due to spatial non-uniformity [31]. Lettuce plants were irrigated daily with approximately 100–200 mL of the appropriate NS until harvest. 

Plants were sampled 35 and 53 (final harvest) days after transplanting (DAT). At each sampling time, leaves of each plant were separated from roots and used for subsequent determinations.

### 2.4. Determinations

Leaves or roots were used for the evaluation of AMF colonization, growth parameters, leaf antioxidant capacity and concentrations of chlorophylls, carotenoids and total phenols. Nitrate content in lettuce leaves (NO_3_^−^) and mineral content of both leaves and roots were determined only at final harvest (53 DAT). Each determination was performed on four replicates per treatment.

#### 2.4.1. AMF Colonization and Growth Parameters

Root colonization was assessed at transplant, and 35 and 53 days later. Percentages of AMF colonization were determined under a dissecting microscope using the gridline intersect method [32], after clearing and staining plant roots with Trypan blue in lactic acid (0.05% *w*/*v*).

Leaf area, leaves, and root fresh (FW) and dry weight (DW) were determined at 35 and 53 DAT. Dry weight was measured after drying in a ventilated oven at 70 °C until constant weight. Leaf area was measured using a digital planimeter (MK2, Delta-T Devices, Cambridge, UK).

#### 2.4.2. Leaf Content of Chlorophylls, Carotenoids, Total Phenols, and Antioxidant Capacity

Five foliar disks (12 mm diameter, 0.5 g approximately) from distinct leaves were sampled for each replicate plant and extracted with 5 mL of methanol for the determination of the leaf content of chlorophylls (chlorophyll a and chlorophyll b), carotenoids, total phenols, and the antioxidant capacity. Samples were sonicated 15 min in ice bath four times and stored overnight at −20 °C. After separation of the supernatant, the extraction was repeated with 5 mL of fresh methanol. The two supernatant aliquots were pooled and, after proper dilution with methanol, the absorbance of the extracts was read at 665.2, 652.4, and 470 nm (Shimadzu UV-1280, Tokyo, Japan). The leaf concentrations of chlorophylls and carotenoids were calculated according to Lichtentahler and Buschmann [33] and expressed as μg g^−1^ FW.

Total phenols were measured in the same methanol extracts using the Folin–Ciocalteau reagent [34]. The spectrophotometric measurements were carried out at 765 nm and the total phenol content was calculated using a calibration curve prepared with standard solutions (0, 50, 100, 150 and 250 mg L^−1^) of gallic acid (GA); values were expressed as gallic acid equivalents per g FW (mg GAE g^−1^ FW)

The antioxidant capacity was determined by the Ferric Reducing Antioxidant Power (FRAP) assay according to Benzie and Strain [35]. The following solutions were mixed in a spectrophotometric cuvette: 0.25 M acetate buffer pH 3.6 (2.0 mL); FRAP reagent (900 μL) containing 2 mM ferric chloride and 1 mM TPTZ (2,4,6-tripyridyl-s-triazine). A calibration curve was prepared with standard solutions of ferrous ammonium sulphate up to 1000 μM concentration. The absorbance was detected at 593 nm and the results were expressed as μmol Fe (II) g^−1^ FW.

#### 2.4.3. Leaf and Root Mineral Content

Plant samples were dried for 5 days in a 70 °C oven to constant weight, then they were ground in a mortar to a powder. The oven-dried samples were wet digested in a mixture of nitric and perchloric acids (5:2 *v*/*v*) at 230 °C for 1 h and the elements K, Ca, Mg, Na, Fe, Mn, Cu, and Zn were quantified by atomic absorption spectrometry (Varian Model Spectra AA240 FS, Melbourne, Australia). Phosphorus was determined by spectrophotometry using the molybdenum blue method [36], and N was determined by micro-Kjeldahl procedure [37] after sample digestion with sulfuric acid (H_2_SO_4_).

Leaf content of soluble nitrates was also determined by extracting 100 mg of powdered dry samples with 20 mL distilled water on an orbital shaker at room temperature for two hours; the aqueous extract was then analyzed as described [38] and leaf nitrate concentration was expressed as mg NO_3_^−^kg^−1^ FW.

#### 2.4.4. Leaf Gas Exchanges

Net photosynthetic rate (Pn), stomatal conductance (gs), intercellular CO_2_ concentration (Ci), and transpiration rate (E) were determined in fully expanded leaves using a portable instrument (CIRAS-2 photosynthesis system, PP Systems, Amesbury, MA, USA) at 250 μmoL m^−2^ s^−1^ of PPFD, 400 ppm CO_2_ concentration, 65% relative humidity, and 25 °C leaf temperature, which was close to the growth chamber temperature. Intrinsic water use efficiency (WUEi) was calculated as the ratio of Pn to gs.

### 2.5. Statistical Analysis

Data were analyzed by the analysis of variance (ANOVA) and mean values of 4 replicates were separated using the Least Significant Differences (LSD; *p* = 0.05). Statistical analysis was performed using the software Statgraphics Centurion XV.II (Manugistic Co., Rockville, MD, USA).

As the interaction between sampling time and treatment was not significant for all the measured quantities, only the main effects are reported in the next section.

## 3. Results

### 3.1. Mycorrhizal Colonization and Plant Growth, Mineral Content, and Leaf Gas Exchange 

Root colonization in AMF inoculated plants (LPM) was 35.1 ± 4.9% at transplant, 21.8 ± 2.5% at the intermediate sampling, and 20.5 ± 3.2% at the final harvest. Control plants (both LPC and HPC) were not colonized throughout the whole experiment. 

Leaf FW and DW, root DW, and leaf area were greater at 53 DAT than at 35 DAT, as expected, while there were no significant differences between the two sampling times as regards the root/shoot DW ratio (Table 1). Leaf FW and DW (Table 1) showed no significant differences between HPC and LPM plants, while they were significantly lower in LPC plants with respect to other plants (Table 1). On the contrary, LPM plants showed greater root DW compared with HPC and LPC plants. Root/shoot DW ratio was significantly greater in LPC than in HPC, with intermediate value in LPM. Conversely, leaf area was significantly greater in LPM than in LPC, with intermediate value in HPC.

At the final sampling time (53 DAT), leaf content of N and P was significantly lower in LPC plants than in HPC and LPM plants (Table 2). Leaf Mg content was significantly lower in non-mycorrhizal controls, LPC and HPC, than in LPM plants (Table 2). Leaf contents of other mineral elements were not significantly affected by treatments (Table 2). 

In lettuce roots, N content was not affected by treatments, while the P content was statistically higher in LPM plants than in LPC and HPC plants. 

As for the other elements, only root contents of Fe and Cu were statistically affected by treatments, with higher values of Fe in LPM plants than in LPC and HPC plants, and lower values of Cu in HPC plants than in LPC and LPM plants (Table 3).

**Figure 1:**
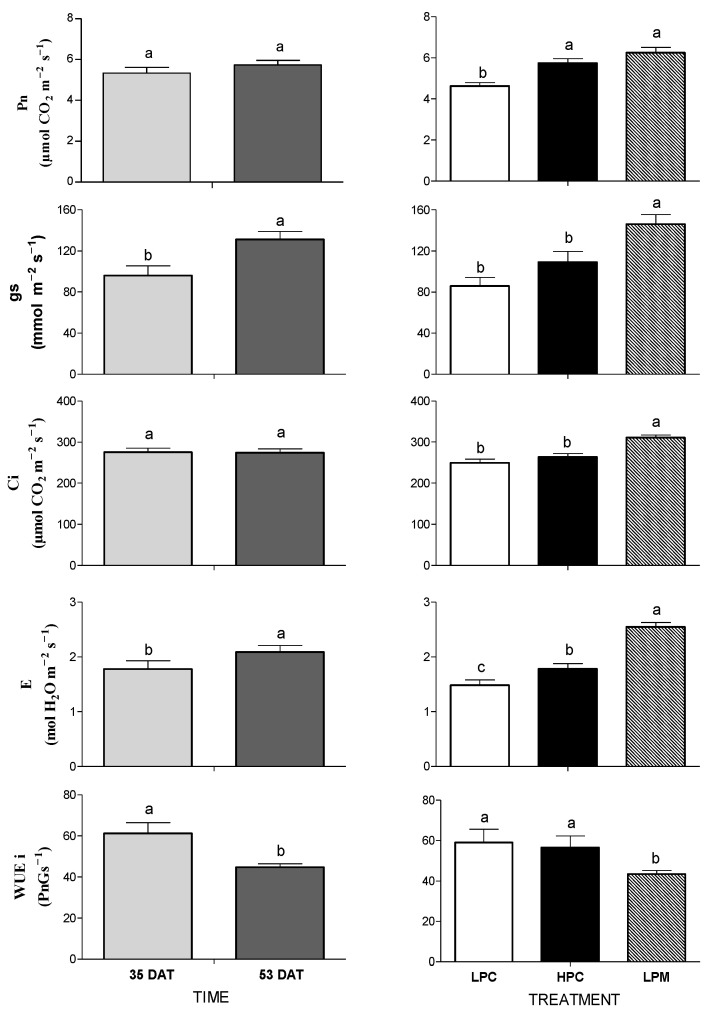
Leaf gas exchanges (Pn = net photosynthetic rate, gs = stomatal conductance, Ci = intercellular CO_2_ concentration, E = transpiration rate, WUEi = intrinsic water use efficiency) determined at 35 and 53 days after transplant (DAT) in lettuce plants non-inoculated or inoculated with the arbuscular mycorrhizal fungus *Funneliformis mosseae* and grown hydroponically with different P concentration in the nutrient solution (LPC = Low Phosphorus Control; HPC = High Phosphorus Control; LPM = Low Phosphorus Mycorrhizal). Mean values (n = 4) ± SE followed by different letters are statistically different (*p* < 0.05) according to the LSD test. Only main effects are shown, as interaction was not significant.

Likewise, growth traits and leaf gas exchange parameters were not affected by the interaction between sampling time and treatments, so only the main effects are shown herein (Figure 1; all results are shown in Table S1). Time had different effects, since significant increase in leaf gs and E were observed from 35 DAT to 53 DAT, while Pn and Ci were not affected by sampling time and WUEi decreased significantly at 53 DAT. Treatments significantly affected all the measured variables, with LPM plants showing higher values of leaf gs, Ci, and E than non-mycorrhizal controls, and higher values of Pn than LPC plants. On the contrary, LPM plants showed significantly lower values of WUEi than non-mycorrhizal controls (Figure 1).

### 3.2. Content of Pigments, Total Phenols, Antioxidant Capacity, and Nitrates of Leaves

Pigment and total phenol content and antioxidant capacity showed significantly higher values in LPM plants than in non-mycorrhizal plants (Table 4). 

**Figure 2:**
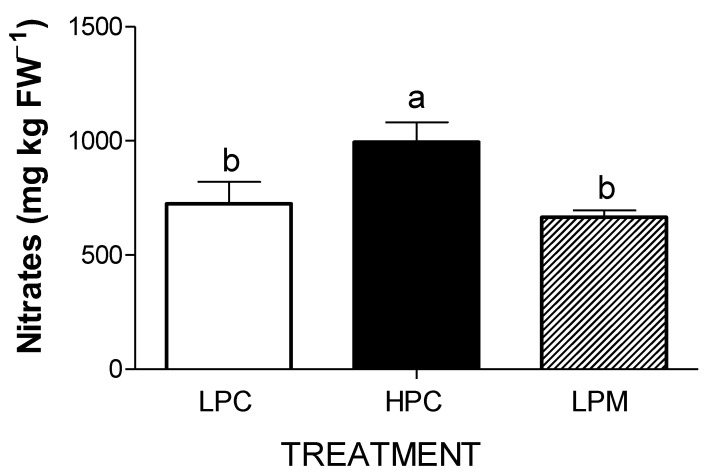
Leaf nitrate content at 53 DAT in lettuce plants non-inoculated or inoculated with the arbuscular mycorrhizal fungus *Funneliformis mosseae* and grown hydroponically with different P concentration in the nutrient solution (LPC = Low Phosphorus Control; HPC = High Phosphorus Control; LPM = Low Phosphorus Mycorrhizal). Mean values (n = 4) ± SE followed by different letters are statistically different (*p* < 0.05) according to the LSD test.

Leaf nitrate content, measured only at final harvest (53 DAT), was significantly higher in HPC plants than in LPC and LPM plants (Figure 2).

## 4. Discussion

Data presented in this work clearly show that the nutritional value of soilless grown lettuce of cv. Salinas was improved when AMF-inoculated and grown at low P concentration compared with non inoculated plants grown at low or optimal P concentrations. At the same time, mycorrhizal plants performed like plants grown at the higher P concentration.

### 4.1. Mycorrhizal Colonization and Plant Growth, Mineral Content, and Leaf Gas Exchange

Mycorrhizal lettuce plants showed a colonization of approximately 20%, which was lower than that found in other studies with *L. sativa* var. *capitata* grown in different conditions and inoculated with different AMF [22,39]. This large variability is known for lettuce genotypes such as those belonging to *Lactuca sativa* var. *crispa*, where different levels of root colonization were reported ranging from 10–15% in cv. Eluarde and Panisse [40] to 45–65% in cv. Grand Rapids and Lollo Bionda [41], grown in different substrates and with different AMF. The degree of root colonization depends on the interaction between host and fungal genotypes and is modulated by environmental conditions, in particular by temperature [42,43,44].

Other important factors which can explain the low level of colonization attained in this work are the P concentration of the NS and the high level of water content of the soilless substrate. Actually, it is well known that high P supply reduces root colonization levels [45,46], possibly in concert with other nutrients [47]. In addition, when substrate moisture increases, root colonization decreases [48,49]. In our work, the P concentration in the NS was adjusted to avoid both an excessive reduction in colonization and a nutritional stressful condition. Other authors were able to grow *L. sativa* var. *capitata* plants in a hydroponic system (nutrient film technique or NFT) with the aim of producing fungal biomass, maintaining a high level of colonization using NS with 20–80 μΜ P, however they obtained a very low plant biomass [50]. In another study using non-mycorrhizal *L. sativa* var. *capitata* plants, reducing nutrient supply led to a 14% (at half-strength NS) to 38% (at quarter-strength NS) decrease in fresh biomass [9].

However, at the level of colonization obtained in our system, LPM plants showed an increase in all the growth traits compared with LPC plants, while no differences were observed between LPM plants and HPC plants. During the experiment no evident symptoms of nutrient deficiency (e.g., leaf chlorosis or necrosis) were observed in all the plants, and leaf content of most macroelements and trace elements were similar and within the adequate ranges reported for lettuce [51].

More specifically, leaf content of both N and P was significantly higher in HPC and LPM plants than that in LPC plants, showing that AMF inoculation allowed the plants to cope with the reduction in P in the NS (Table 2) with no important effect on N uptake.

In our work, mycorrhizal plants showed significantly higher levels of Mg in the leaves (Table 2). Magnesium plays an important role in plant cells, since it appears in the center of the chlorophyll molecule and many enzymatic reactions require Mg as a co-factor. The higher level of chlorophylls in mycorrhizal lettuces confirms this. A higher concentration of Mg in inoculated plants was found in lettuce [22], cucumber [52], and tomato [53]. Moreover, a higher content of P, Fe, and Cu was found in roots of inoculated lettuces (Table 3).

Our results regarding leaf gas exchanges are in agreement with previous findings, which demonstrated the positive effects of AMF on leaf photosynthesis [54,55,56,57] and suggest that LPM plants were metabolically similar to HPC plants and more efficient than LPC. These data, referred to primary metabolism, support the higher growth observed in LPM with respect to LPC plants. The LPM plants showed lower values of WUEi in comparison with LPC and HPC plants as a result of higher gs than Pn in LPM plants. This is in contrast with other studies that showed an increase in WUEi after AMF inoculation [55,58]. Recently, Ref.[59] found lower WUEi in AMF-inoculated wheat (*Triticum aestivum* L.) plants in comparison with non-inoculated plants. However, other authors [60] showed that the plant WUEi was reduced, enhanced, or not influenced by AMF colonization because it is strictly associated to the symbiosis between the plant and the AMF species applied. 

In conclusion, these findings are in agreement with those obtained in cucumber [57] and tomato [61] and support the role of AMF in plant response to a reduced P supply, avoiding a loss of production. Some authors recommended that the P concentration of the NS should be reduced up to 50% when AMF inoculated plants are cultivated in an ebb–flow system [62].

### 4.2. Content of Pigments, Total Phenols, Antioxidant Capacity, and Nitrates of Leaves

Mycorrhizal plants not only showed better or similar growth than non-inoculated plants, but their leaves contained more chlorophylls, carotenoids, and total phenols, and had a stronger antioxidant capacity.

Arbuscular mycorrhizal fungi are known to facilitate host plants to grow under stressful conditions by enhancing leaf photosynthesis and root water uptake and by acting on hormonal biosynthesis and secondary metabolic pathways [18,63]. The higher mineral uptake in mycorrhizal plants was significantly correlated with a higher content of bioactive compounds, thus suggesting a close relationship among the modulation of nutrient uptake by mycorrhizal symbionts and the biosynthesis of health-promoting molecules by the host [64], which was not observed in HPC plants compared with LPC plants. 

In our study there was a high statistically significant correlation between the FRAP index and the leaf content of total phenolic compounds (r = 0.654, *p* = 0.001, n = 24). Some authors showed a significant rise in phenols and antioxidant activity in lettuce plants (red and green leaf *L. sativa* var. *crispa*) inoculated with R. *irregulare* compared to non-mycorrhizal plants [40].

Previous reports on lettuce inoculated with AMF showed an increase in plant growth and a better leaf nutritional quality due to a greater content of both vitamins and pigments, although plants were supplied with 1.3 mM P and the substrate contained a substantial quantity of peat, which conveys further P and organic matter [21]. In another work with lettuce grown in NFT, plants treated with a commercial microbial inoculant showed a better growth performance and a higher content of anthocyanin than non-inoculated plants [65].

In addition to the content of secondary metabolites, the quality of lettuce leaves is determined by the content of nitrates, which can have a harmful effect on human health [66]. For this reason, the EU regulation 1258/2011 fixed the upper limits for iceberg lettuce, such as cv. Salinas used in this experiment, to 2.000 mg kg^−1^ in open field and 2.500 mg kg^−1^ under cover [67]. In our work, nitrate leaf content was always well below the EU regulation limits, and was lower in LPM and LPC than in HPC plants. These findings agree with those reported by [9,66,68,69], who concluded that nitrate accumulation in lettuce is strongly correlated with the composition of NS and influenced by phosphate nutrition. As reported in [9], nitrate content decreased by 34.6% in butterhead lettuce grown with quarter-strength nutrient solution in comparison with the full- and half-strength nutrient solution. Replacing the nutrient solution with tap water two days before harvest reduced the nitrate concentration by up to 56% in *Lactuca sativa* var. *acephala* [69].

## 5. Conclusions

Our data show that mycorrhizal inoculation in soilless culture, when symbionts are reciprocally compatible as in this case, can increase the nutritional quality of *L. sativa* var. *capitata* and allow the cultivation with reduced phosphorus concentration in the nutrient solution with no yield loss, thus mitigating the environmental impact associated with this growing system.

The utilization of a lettuce cultivar whose genome has been sequenced [70] may allow further studies investigating plant/fungus gene expression profiles and biochemical mechanisms involved in symbiosis beneficial effects aimed at exploiting mycorrhizal fungi as biostimulants.

## Figures and Tables

**Table 1:** Growth parameters determined at 35 and 53 days after transplant (DAT) in lettuce plants non-inoculated or inoculated with the arbuscular mycorrhizal fungus *Funneliformis mosseae* and grown hydroponically with different phosphorus (P) concentration in the nutrient solution (LPC = Low Phosphorus Control; HPC = High Phosphorus Control; LPM = Low Phosphorus Mycorrhizal).

Sampling Time (DAT)	Treatment	Leaf FW (g Plant^−1^)	Leaf DW (g Plant^−1^)	Root DW (g Plant^−1^)	Root/Shoot DW Ratio	Leaf Area (cm^2^ Plant^−1^)
35	LPC	66.6 ± 6.9	2.94 ± 0.36	0.48 ± 0.17	0.17 ± 0.02	719.1 ± 80.9
35	HPC	104.8 ± 3.5	4.93 ± 0.39	0. 65 ± 0.07	0.13 ± 0.01	1233.3 ± 142.6
35	LPM	155.8 ± 14.4	7.32 ± 0.58	0.88 ± 0.05	0.12 ± 0.01	1674.5 ± 172.7
53	LPC	135.6 ± 10.3	5.46 ± 0.36	0. 93 ± 0.07	0.17 ± 0.01	1524.9 ± 120.4
53	HPC	221.4 ± 38.7	9.34 ± 1.28	1.00 ± 0.05	0.11 ± 0.01	2471.0 ± 418.6
53	LPM	263.9 ± 29.1	10.86 ± 0.90	1.79 ± 0.24	0.16 ± 0.01	2968.7 ± 328.7
MAIN EFFECTS						
35		109.1 ± 12.1 b	5.06 ± 0.59 b	0.67 ± 0.06 b	0.14 ± 0.01 a	1208.9 ± 137.9 b
53		206.9 ± 21.9 a	8.55 ± 0.84 a	1.24 ± 0.14 a	0.15 ± 0.01 a	2321.6 ± 244.3 a
	LPC	101.1 ± 14.2 b	4.20 ± 0.53 b	0.71 ± 0.09 b	0.17 ± 0.01 a	1122.0 ± 166.5 b
	HPC	163.1 ± 28.4 a	7.13 ± 1.04 a	0.82 ± 0.08 b	0.12 ± 0.01 b	1852.1 ± 310.8 ab
	LPM	209.9 ± 25.4 a	9.09 ± 0.83 a	1.33 ± 0.21 a	0.14 ± 0.01 ab	2321.6 ± 298.9 a
	ANOVA (ns, not significant; **, significant at 1%; ***, significant at 0.1%)					
Time		***	***	***	ns	***
Treatment		***	***	***	**	***
Interaction		ns	ns	ns	ns	ns

Mean values (n = 4) ± standard error (SE) followed by different letters are statistically different (*p* < 0.05) according to the LSD test.

**Table 2:** Leaf mineral content determined at 53 DAT in lettuce plants non-inoculated or inoculated with the arbuscular mycorrhizal fungus *Funneliformis mosseae* and grown hydroponically with different P concentration in the nutrient solution (LPC = Low Phosphorus Control; HPC = High Phosphorus Control; LPM = Low Phosphorus Mycorrhizal).

Treatment	N	P	K	Na	Ca	Mg	Fe	Cu	Mn	Zn
	(g kg^−1^ DW)						(mg kg^−1^ DW)			
LPC	35.7 ± 3.0 b	3.2 ± 0.2 b	79.7 ± 5.9 a	4.8 ± 0.8 a	16.3 ± 1.4 a	3.1 ± 0.3 b	119.6 ± 19.7 a	10.7 ± 0.6 a	111.5 ± 10.2 a	48.3 ± 2.2 a
HPC	41.5 ± 0.8 a	6.5 ± 0.3 a	70.5 ± 4.7 a	6.0 ± 0.9 a	15.2 ± 1.2 a	2.4 ± 0.3 b	174.5 ± 58.0 a	9.1 ± 0.5 a	111.9 ± 4.9 a	49.1 ± 2.1 a
LPM	42.7 ± 0.5 a	6.4 ± 0.1 a	71.2 ± 6.0 a	4.9 ± 0.4 a	16.9 ± 2.0 a	4.2 ± 0.2 a	127.3 ± 7.8 a	10.7 ± 0.5 a	129.6 ± 11.8 a	76.5 ± 28.0 a
	ANOVA (ns, not significant; **, significant at 1%; ***, significant at 0.1%)									
Treatment	**	***	ns	ns	ns	**	ns	ns	ns	ns

The mean values (n = 4) ± SE followed by different letters are statistically different (*p* < 0.05) according to the LSD test.

**Table 3:** Root mineral content determined at 53 DAT in lettuce plants non-inoculated or inoculated with the arbuscular mycorrhizal fungus *Funneliformis mosseae* and grown hydroponically with different P concentration in the nutrient solution (LPC = Low Phosphorus Control; HPC = High Phosphorus Control; LPM = Low Phosphorus Mycorrhizal).

Treatment	N	P	K	Na	Ca	Mg	Fe	Cu	Mn	Zn
	(g kg^−1^ DW)						(mg kg^−1^ DW)			
LPC	19.3 ± 5.0 a	3.3 ± 0.2 b	27.9 ± 2.0 a	8.4 ± 2.3 a	12.3 ± 1.0 a	12.9 ± 0.6 a	1616.2 ± 199.2 b	12.5 ± 1.2 a	267.9 ± 33.7 a	77.6 ± 6.9 a
HPC	17.7 ± 1.6 a	3.3 ± 0.4 b	26.1 ± 1.7 a	11.7 ± 1.5 a	10.9 ± 0.9 a	15.0 ± 2.9 a	1276.4 ± 173.8 b	7.7 ± 1.1 b	247.3 ± 29.2 a	56.5 ± 4.5 a
LPM	22.9 ± 2.7 a	4.5 ± 0.3 a	32.2 ± 2.9 a	13.4 ± 1.5 a	12.9 ± 1.3 a	16.3 ± 1.2 a	2993.5 ± 554.5 a	13.6 ± 1.4 a	301.7 ± 32.6 a	70.8 ± 4.3 a
	ANOVA (ns, not significant; *, significant at 5%)									
Treatment	ns	*	ns	ns	ns	ns	*	*	ns	ns

The mean values (n = 4) ± SE followed by different letters are statistically different (*p* < 0.05) according to the LSD test.

**Table 4:** Leaf content of chlorophylls, carotenoids, total phenols, and antioxidant capacity (FRAP index) determined at 35 and 53 DAT in lettuce plants non-inoculated or inoculated with the arbuscular mycorrhizal fungus *Funneliformis mosseae* and grown hydroponically with different P concentration in the nutrient solution (LPC = Low Phosphorus Control; HPC = High Phosphorus Control; LPM = Low Phosphorus Mycorrhizal).

Sampling Time (DAT)	Treatment	Chlorophylls (μg g^−1^ FW)	Carotenoids (μg g^−1^ FW)	Total Phenols (mg GAE g^−1^ FW)	FRAP (μmol Fe(II) g^−1^ FW)
35	LPC	627.25 ± 14.60	95.14 ± 5.58	2.82 ± 0.23	15.95 ± 0.96
35	HPC	608.80 ± 38.77	95.18 ± 7.80	2.65 ± 0.19	16.07 ± 1.25
35	LPM	845.55 ± 40.26	131.32 ± 2.89	3.49 ± 0.09	21.32 ± 1.51
53	LPC	593.57 ± 46.04	91.95 ± 4.81	2.16 ± 0.08	16.90 ± 0.69
53	HPC	663.04 ± 24.06	93.68 ± 4.37	2.08 ± 0.26	14.15 ± 1.55
53	LPM	888.93 ± 49.48	126.00 ± 6.59	2.98 ± 0.17	20.97 ± 1.20
MAIN EFFECT					
35		693.87 ± 36.80 a	107.21 ± 5.96 a	2.99 ± 0.14 a	17.78 ± 1.00 a
53		715.18 ± 43.74 a	103.88 ± 5.48 a	2.41 ± 0.14 b	17.34 ± 1.05 a
	LPC	610.41 ± 23.25 b	93.55 ± 3.46 b	2.49 ± 0.17 b	16.42 ± 0.58 b
	HPC	635.92 ± 23.48 b	94.43 ± 4.15 b	2.36 ± 0.15 b	15.11 ± 0.99 b
	LPM	867.24 ± 30.65 a	128.66 ± 3.48 a	3.24 ± 0.13 a	21.14 ± 0.89 a
ANOVA (ns, not significant; **, significant at 1%; ***, significant at 0.1%)					
Time		ns	ns	***	ns
Treatment		***	***	***	***
Interaction		ns	ns	ns	ns

Mean values (n = 4) ± SE followed by different letters are statistically different (*p* < 0.05) according to the LSD test.

## Data Availability

Not applicable.

## Supplementary Materials

The following supporting information can be downloaded at: https://www.mdpi.com/article/10.3390/foods11223612/s1, Table S1: Gas exchange parameters; net photosynthetic rate (Pn), stomatal conductance (gs), intercellular CO_2_ concentration (Ci), transpiration rate (E), and intrinsic water use efficiency (WUEi; Pn/gs) measured in lettuce leaves during the experiment (1st measurement and 2nd measurement 35 DAT and 53 DAT, respectively).
