# Supplementary material for: Arbuscular Mycorrhizal Fungi Increase Nutritional Quality of Soilless Grown Lettuce while Overcoming Low Phosphorus Supply
Source: Foods. 2022 Nov 12;11(22):3612. doi: 10.3390/foods11223612 (PMC9689368; doi:10.3390/foods11223612)
Supplement: Supplementary file 1 [file foods-11-03612-s001.zip › foods-2000183-supplementary.pdf]

Table S1. Gas exchange parameters; net photosynthetic rate (Pn), stomatal conductance (gs), intercellular CO_2 concentration (Ci), transpiration rate (E) and intrinsic water user efficiency (WUEi; Pn/gs) measured in lettuce leaves during the experiment (1st measurement and 2nd measurement respectively 35 DAT and 53 DAT) LPC = Low Phosphorus Control; HPC = High Phosphorus Control; LPM = Low Phosphorus Mycorrhizal.

Sampling time (DAT)	Treatment	Pn (μmol CO_2 m^{-2} s^{-1})	gs (mmol m^{-2} s^{-1})	Ci (μmol mol^{-1})	E (mol H_2O m^{-2} s^{-1})	WUEi (μmol CO_2 mol^{-1} H_2O)
35 DAT	LPC	4.40 ± 0.26	66.60 ± 9.21	250.60 ± 13.78	1.32 ± 0.15	71.41 ± 10.76
35 DAT	HPC	5.70 ± 0.41	85.00 ± 8.41	263.20 ± 12.26	1.56 ± 0.13	69.08 ± 6.31
35 DAT	LPM	5.92 ± 0.46	136.60 ± 11.52	312.40 ± 9.18	2.46 ± 0.11	43.56 ± 1.43
53 DAT	LPC	4.84 ± 0.16	105.00 ± 7.40	278.43 ± 14.72	1.64 ± 0.11	46.78 ± 1.82
53 DAT	HPC	5.78 ± 0.24	133.40 ± 8.12	296.35 ± 12.44	2.00 ± 0.04	44.20 ± 3.87
53 DAT	LPM	6.58 ± 0.20	155.60 ± 13.21	370.80 ± 12.74	2.64 ± 0.11	43.31 ± 3.13
			MAIN EFFECT			
35 DAT		5.34 ± 0.27 a	96.07 ± 9.50 b	275.40 ± 9.55 a	1.78 ± 0.15 b	61.35 ± 5.14 a
53 DAT		5.74 ± 0.22 a	131.33 ± 7.66 a	274.07 ± 9.25 a	2.09 ± 0.12 a	44.77 ± 1.68 b
	LPC	4.63 ± 0.16 b	85.80 ± 8.48 b	249.60 ± 8.98 b	1.48 ± 0.10 c	59.09 ± 6.58 a
	HPC	5.74 ± 0.23 a	109.20 ± 10.30 b	263.90 ± 8.23 b	1.78 ± 0.10 b	56.64 ± 5.71 a
	LPM	6.25 ± 0.28 a	146.10 ± 9.33 a	310.70 ± 7.00 a	2.55 ± 0.08 a	43.44 ± 1.71 b
ANOVA (ns, not significant; *, significant at 5%; **, significant at 1%; ***, significant at 0.1%)						
Time		***	***	***	ns	***
Treatment		***	***	***	**	***
Interaction		ns	ns	ns	ns	ns

Mean values (n = 4) ± SE followed by different letters are not statistically different (P <0.05) according to the LSD test.
